# At the Beginning of the End and in the Middle of the Beginning: Structure and Maintenance of Telomeric DNA Repeats and Interstitial Telomeric Sequences

**DOI:** 10.3390/genes10020118

**Published:** 2019-02-05

**Authors:** Anna Y. Aksenova, Sergei M. Mirkin

**Affiliations:** 1 Laboratory of Amyloid Biology, St. Petersburg State University, 199034 St. Petersburg, Russia; 2 Department of Biology, Tufts University, Medford, MA 02421, USA

**Keywords:** telomeric repeats, telomeres, genome stability, microsatellites, repeat expansion, alternative lengthening of telomeres

## Abstract

Tandem DNA repeats derived from the ancestral (TTAGGG)n run were first detected at chromosome ends of the majority of living organisms, hence the name telomeric DNA repeats. Subsequently, it has become clear that telomeric motifs are also present within chromosomes, and they were suitably called interstitial telomeric sequences (ITSs). It is well known that telomeric DNA repeats play a key role in chromosome stability, preventing end-to-end fusions and precluding the recurrent DNA loss during replication. Recent data suggest that ITSs are also important genomic elements as they confer its karyotype plasticity. In fact, ITSs appeared to be among the most unstable microsatellite sequences as they are highly length polymorphic and can trigger chromosomal fragility and gross chromosomal rearrangements. Importantly, mechanisms responsible for their instability appear to be similar to the mechanisms that maintain the length of genuine telomeres. This review compares the mechanisms of maintenance and dynamic properties of telomeric repeats and ITSs and discusses the implications of these dynamics on genome stability.

## 1. Telomeres

### 1.1. Composition and Diversity of Telomeric Repeats

The ends of chromosomes, called telomeres, are natural protective caps that perform two important functions: they prevent chromosomes from end-to-end fusions and from their fusion to accidental double-strand breaks (DSBs) [1,2,3,4] and preclude recurrent DNA loss occurring during replication [5,6,7]. Thus, the functioning of replication and repair machinery at telomeres is strictly regulated to assure that their length is maintained. This is achieved by recruitment of telomerase or via telomerase-independent mechanisms [7,8]. Telomeres and subtelomeres are exceedingly enriched in repetitive DNA: most eukaryotes possess chromosomal ends made up of short (5–8 bp) species-specific simple tandem repeats which are commonly called telomeric repeats [9,10]. Telomeric repeats in all eukaryotic kingdoms share similarity and appear to originate from the same ancestral grounding telomere repeat. The most widespread telomeric motif is the hexanucleotide TTAGGG repeat motif which is characteristic for all Metazoans [11]. It is common for telomeres in all vertebrates and it is also found in *Echinodermata* and *Cephalochordata* [11]. TTAGGG repeats are the building blocks of telomeres in some invertebrates, including *Lepidoptera* species and flat worms *Mollusca* and *Annelida* [12]. This motif is also present at telomeres in some protozoa (*Trypanosoma*), slime molds and in the major fungal classes *Ascomycota* and *Basidiomycota*. At the same time, several fungi genera represent very complex (*Candida* and *Kluyveromyces*) or irregular (*Saccharomyces* and *Shizosaccharomyces*) telomeric runs [9]. Many lineages of the insect tree and non-insect arthropods are characterized by the pentanucleotide TTAGG telomeric repeat motif that likely originated from the vertebrate-type TTAGGG repeat [12,13]. Telomeric repeats in most *Angiosperm and Gymnosperm* plants as well as in *Bryophytes* are represented by the heptanucleotide motif TTTAGGG originally described in *Arabidopsis thaliana* [14]. Some plants share human-type TTAGGG telomeric repeats or were reported to have unusual or variant telomeric sequences [15]. Telomeric repeats reported in Algae include heptanucleotide TTTAGGG, human-type TTAGGG and octanucleotide TTTTAGGG (*Chlamydomonas reinhardtii*) [9,10,15]. Telomeric repeats found in most protozoan species represent further variation of the vertebrate-like repeat TTAGGG and include such examples as TTGGGG (in *Tetrahymena* and *Glaucoma*) or TTTTGGGG (*Oxytricha* and *Euplotes*) [10].

Telomeric repeats may be regular (such as TTAGGG in humans and most vertebrates or TTGGGG in *Tetrahymena*) or irregular (such as G_1–3_T in yeast *Saccharomyces cerevisiae*), and their actual copy number at the end of chromosome may vary from as few as 2–3 repeats in hypotrichous ciliates, such as *Oxytricha*, to thousands of copies in mammals and the total length of a telomere can reach more than 100 kb in mice and up to 2 Mb in chicken [10,16]. Notably, one of the DNA strands of telomeric repeats is enriched in G residues. Importantly, this strand runs 5’→3’ toward the chromosome end and it is longer than its C-rich counterpart, forming a 3’-overhang at the end of a chromosome. This G-rich overhang serves as a substrate for telomerase, which adds telomeric repeats to its terminus prior to each replication round. The length of the G-rich overhang fluctuates depending on the cell cycle stage. For instance, budding yeast’s telomeres are very short and are composed of irregular repeats with a double-stranded region of on average 300±75 bp and a short single-stranded G-rich overhang whose length is maintained around 12–15 nt throughout most of the cell cycle, although significantly longer G-rich overhangs are generated during DNA replication [17]. Human telomeres may vary from 5 to 15 kb on average in size whereas the single-stranded G-rich overhang varies from a dozen to several hundreds of nucleotides [18,19,20,21]. Despite variations in length and sequence composition, telomeres of different species are all capable of end protection and suppression of DNA damage repair. Importantly, the sequence variation reflects diversity in the mechanisms that have evolved to protect chromosomal ends from degradation and unwarranted repair. 

Note that while in most organisms, telomeres are built of TTAGGG-like repeats, some insect species belonging to various orders including *Diptera, Coleoptera, Neuroptera, Palaeoptera*, etc., eliminated these repeats altogether by evolving alternative telomere structures [13]. A striking example is the structure of *Drosophila* telomeres composed of tandem non-LTR retrotransposons [22,23,24,25]. This mechanism could result from the accumulation of telomeric retrotransposons that target TTAGG and variant TCAGG repeats in a sequence-specific manner [26,27].

### 1.2. DNA Structures Formed by Telomeric Repeats In Vitro and In Vivo

High asymmetry in the G-content between the strands has apparent biological relevance. G-rich overhangs were proposed to form a polymorphic class of four-stranded G4-DNA structures characterized by planar G-quartets stabilized by Hoogsteen hydrogen bonds stacked upon each other [28,29,30,31]. G4-DNA is remarkably stable owing to strong π-π–stacking interactions between adjacent G-quartets. Consequently, the number of stacked quartets define the stability of the whole structure. Since the G-rich strand of telomeric repeats usually contains repetitive clusters of 2–4 guanines, it can form relatively stable structures with 2–4 G-quartets stacked together. G4-DNA polymorphism has been extensively studied in vitro and it appears to depend on many factors [31,32,33]. Formation of G-quadruplexes can involve one molecule (intramolecular G-quadruplexes) or several molecules (intermolecular G-quadruplexes). Also, the relative orientation of DNA strands differes between different G4 structures. 

These G4 variants are speculated to have biological roles ranging from the inhibition of telomerase activity [34] and protection of chromosome ends [35] to mediating chromosome arrangement during meiosis and mitosis [30,36]. Formation of G-quadruplex structures in vivo at telomeres was subsequently visualized by immunostaining with specific high-affinity single chain antibodies [37,38,39]. Moreover, the position of G-quadruplexes was mapped at high resolution in genomic DNA from human cancer cells and yeast cells [38,39,40]. Note that both visualization and mapping experiments have shown the formation of G-quadruplexes at chromosomal ends as well as outside telomeric regions and their association with DNA replication.

The complimentary C-rich strand of telomeric DNA is also able to form four-stranded DNA structures. This becomes possible because cytosines in the protonated form can form pairs stabilized by three hydrogen bonds [41]. Stretches of two or more cytidines may form intercalated, quadruple-helical structures under acidic conditions: the so-called i-motifs [42]. These structures can be formed by association of different molecules (tetramers or dimers of two strands containing two distinct C-rich stretches) or by intramolecular folding of a single strand with four cytidine stretches [43]. 

**Figure 1:**
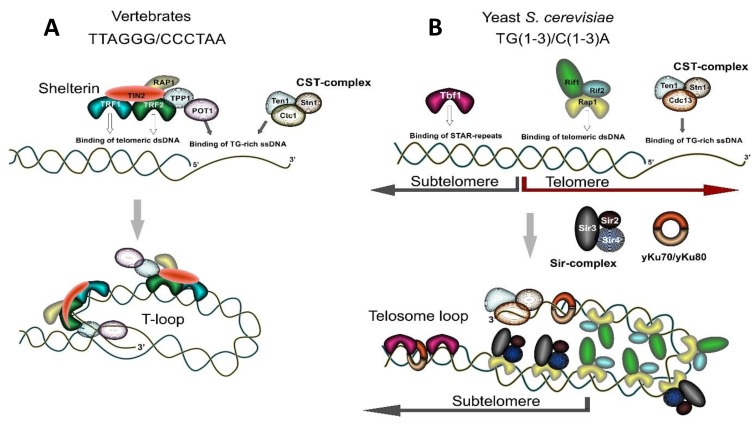
Abridged scheme of telomere organization in vertebrates and in budding yeast showing major telomere-specific protein. (**A**) Schematic representation of the shelterin complex and CST-complex bound to a vertebrate telomere. TRF1 and TRF2 proteins bind to the double-stranded telomeric DNA, POT1/TPP1 and CST-complex bind to the G-rich single-stranded telomeric overhang. Invasion of the G-rich overhang into the double-stranded telomeric repeat transforms this protein-DNA complex into a lariat-like structure called a T-loop. Nucleosomes and secondary protein factors implicated in telomere maintenance are not shown. (**B**) Proteins specific to yeast telomeric repeats include Rap1 which binds to the double-stranded telomeric repeat and recruits Rif1 and Rif2 or Sir-proteins, and CST-complex bound to the G-rich single-stranded overhang. The Tbf1 protein binds to human telomeric repeats, called STAR-repeats, located at subtelomeric DNA regions. Yeast telomeres can also form a folded-back structure, called telosome, which is stabilized by protein-protein interactions. Sirtuins and Yku70/Yku80 heterodimer participate in telosome formation and telomere maintenance in yeast.

Besides being able to form G4-DNA, electron microscopic analysis revealed that human and murine telomeres can be organized in a large lariat structure, called a t-loop, the size of which correlates with telomere length [44]. Such structures are formed through the looping back of the single-stranded G-rich overhang and its invasion into the duplex telomeric DNA with the formation of a displacement loop (D-loop). T-loops thus represent a stabilized displacement loop (D-loop) at the end of chromosome where the 3’-end of chromosome is sequestered and shielded from the repair and recombination machinery owing to its interaction with specific protein complexes (see Section 1.3). Such protective caps define the natural end of the chromosome and distinguish it from a random DSB. Notably, yeast telomeres are thought to be too short to form T-loops; however, they also form a folded-back structure (a telosome loop) that is stabilized by protein-protein interaction without 3’-end invasion into the duplex DNA [17,45,46,47,48] (Figure 1).

### 1.3. Factors Binding to Telomeric Repeats and Structure of Telomeric Chromatin

#### 1.3.1. Major Protein Factors Binding to Telomeric Repeats

Chromosomal termini appear as broken DNA ends and can be engaged in the repair process if not shielded properly from the DNA damage response (DDR) machinery. The repair of a lesion occurring at telomeres may occur in one of several pathways: non-homologous end joining (NHEJ), homologous recombination (HR), microhomology-mediated end-joining (MHEJ), break-induced DNA replication (BIR), single strand annealing (SSA), etc. [49,50,51,52,53]. The outcome of such repair can be deleterious for the genome: it can lead to fusion of chromosomes and their subsequent breakage in cell division, leading to unequal distribution of genetic material between daughter cells. Once initiated, this process can result in a chain reaction of breakage-fusion-bridge cycles and can ultimately reshape the genome [2,4]. Therefore, chromosomal termini in virtually all eukaryotic organisms from yeast to human possess a threat to genomic stability and must be protected from the repair machinery. In addition, unprotected telomeres elicit a DNA damage signal which normally leads to the cell-cycle arrest, confirming that telomere protection is essential for cell division.

The formation of a protective cap at the end of chromosomes depends on the interaction of several protein factors with telomeric repeats. The six-subunit protein complex called Shelterin is essential for telomere maintenance in vertebrates (Figure 1A). Shelterin consists of TRF1, TRF2, POT1, TPP1, TIN2, and RAP1. Three of these proteins—TRF1, TRF2, and POT1—directly interact with telomeric DNA: TRF1 and TRF2 interact with double-stranded telomeric DNA and POT1 interacts with the single-stranded G-rich tip of a telomeric DNA. TIN2 (TRF1-interacting nuclear factor 2) and RAP1 (Repressor Activator Protein 1) do not directly bind to human telomeric repeats albeit they have contact with other Shelterin proteins. TIN2 interacts with TRF1, TRF2, and TPP1/POT1, while RAP1 is a TRF2-interacting factor. TRF2 has a specific role in facilitating T-loop formation and their resolution [54,55,56,57]. Lower eukaryotes, such as budding yeast, utilize Rap1 protein (the ortholog of human RAP1) for direct recognition of double-stranded telomeric DNA, as they lack the rest of the Shelterin complex components (Figure 1B). The telomere capping function in budding yeast is mainly fulfilled by a protein complex called the CST-complex, composed of Cdc13, Stn1, and Ten1 proteins [58] (see Figure 1B). These proteins participate in the recognition and protection of single-stranded telomeric G-rich overhangs at the very end of yeast chromosomes, as well as in the recruitment of telomerase and DNA polymerase α to deal with the end-replication problem. The CST-complex appears to be universally conserved [58,59,60,61,62,63,64]. Originally described in yeast, it has similar composition in lower and higher eukaryotes: in humans, it is composed of Ctc1, Stn1, and Ten1 proteins where the Ctc1 protein is a functional analog of yeast Cdc13 despite lacking apparent sequence identity [65] (see Figure 1A). 

The CST-complex is also sometimes viewed as a telomere-specific functional analog of the heterotrimeric replication protein A (RPA) complex whose role is to assist the replication fork in transversing hard-to-replicate telomeric heterochromatin [58,59,60,62]. RPA is a highly conserved complex that binds non-specifically to single-stranded DNA and is essential for all pathways where formation and stabilization of ssDNA is required. All RPA subunits contain typical oligonucleotide/oligosaccharide-binding (OB) fold motifs involved in binding of ssDNA. OB-fold motifs are characterized by five β-strands forming a closed β-barrel [66]. Such folds are quite stable and are involved in the binding of various substrates including oligonucleotides and ssDNA [67]. Importantly, that same architecture is used in the CST complex: all three components of CST complex contain OB-fold motifs and CST complex subunits have been proposed to be functional and structural counterparts of the RPA complex subunits [58,59,60,62]. Specific recognition of DNA by the CST-complex is achieved through the interaction of telomeric DNA with OB-fold motifs in Cdc13 and Stn1 proteins.

#### 1.3.2. Distinct RAP1 Function in Lower and Higher Eukaryotes and a Hypothesis about Evolutionary Origin of Telomeres in Budding Yeast

Proteins that bind double-stranded telomeric DNA include TRF1 and TRF2 in humans or Rap1 (Repressor Activator Protein1) in yeast (see Figure 1A,B). As was mentioned above, vertebrates also possess the RAP1 protein—it is a component of the Shelterin complex and is a distant ortholog of yeast Rap1. Yeast Rap1 and human RAP1 share similar architecture: an N-terminal BRCT domain, a DNA-binding domain (consisting of two Myb-related bundles in scRap1 and one in hRAP1) and a C-terminal Rap1-specific protein-interaction domain. The striking difference between these two proteins is that while yeast Rap1 directly interacts with DNA, human RAP1 does not. Yeast monomeric Rap1 recognizes two ACACC sites spaced 8 bp apart and interacts sequence-specifically with telomeric DNA and with telomeric repeats at other genomic sites, where it can play a role in transcription regulation and genome organization [68,69,70,71]. At the same time, human RAP1 uses the TRF2 interface for interaction with telomeric DNA and deletion of TRF2 removes RAP1 from telomeres [72]. Human RAP1 is viewed as an adaptor protein that mediates interaction with other proteins. RAP1 interacts with the Rad50 and Mre11 members of the MRN (MRE11/RAD50/Nbs1) complex, the Ku70 and Ku86 proteins essential for NHEJ and PARP1 (poly-ADP ribose polymerase) [73]. Its essential function includes repression of homology-directed repair (HDR) at telomeres and, although controversial, repression of NHEJ [74,75,76]. Interestingly, human RAP1 has retained its function as a transcription regulator, thus it affects gene expression in many non-telomeric sites across the genome [77,78]. 

The striking difference between Rap1’s role for telomere maintenance in lower and higher eukaryote models generated speculations about the evolutionary origin of telomeres in budding yeast. A common point of view suggests that a yeast ancestor had TTAGGG telomeres and Rap1 bound to a TRF-like module. Then, a change in the telomerase RNA gene triggered telomere repeat divergence and the aquisition of new features by Rap1 [72,74,79]. Consistent with this hypothesis, budding yeast carry low copy arrays of “remnant” TTAGGG repeats positioned within subtelomeric elements Y’ and X [80,81]. These arrays are recognized by a specific protein, Tbf1 (TTAGGG binding factor 1) [80,82] (Figure 1B). Sites of Tbf1 binding are known as STAR elements (for sub-telomeric anti-silencing regions) since they exhibit anti-silencing activities and can counteract Telomere Position Effect (TPE), a phenomenon that manifests as downregulation of transcription of genes adjacent to telomeric sequences [83,84]. Importantly, TRF1 and TRF2 proteins were first identified by homology with the yeast Tbf1 protein, and, together with Tbf1, they form a subfamily of telobox-domain (Myb-related) containing proteins [85]. 

Tbf1 binding can provide Rap1-independent capping of artificial telomeres consisting of TTAGGG repeats or mixed TTAGGG/TG_(1–3)_ repeats [86,87]. Arrays of TTAGGG repeats can serve as a telomere seed in yeast carrying a modified version of the *TLC1* gene in which a portion encoding the template for telomerase reverse transcriptase is substituted with vertebrate telomeric repeats. After extension by the chimeric telomerase such “humanized” telomere protected chromosomal stability at the wildtype level, however it failed to silence expression of the adjacent reporter gene and showed absence of Rap1 and Rif2 proteins accumulation; instead, it did bind Tbf1 and Cdc13 proteins [86,87,88]. Tbf1 is also able to block checkpoint response and regulate telomerase recruitment to DNA ends flanked by TTAGGG repeats in length-dependent way [88,89]. At long TTAGGG telomere seeds, Tbf1 manifests a robust capping function, similar to Rap1—it is able to block 5’-end resection by the MRX complex and checkpoint activation [88]. Similar to Rap1, Tbf1 also functions as a transcriptional regulator and binds to many promoter targets in the genome including small nucleolar RNA promoters [90]. 

#### 1.3.3. Chromatin Organization of Telomeres; Replication and Transcription of Telomeres

In lower eukaryotes such as yeast *S. cerevisiae*, telomeres are a non-nucleosomal nucleoprotein complex [91]. Yeast double-stranded telomeric repeats are bound by the Rap1 protein. Rap1, in turn, recruits Silent Information Regulator, SIR-complex, consisting of the Sir2/Sir3/Sir4 proteins and the Rif1 and Rif2 proteins, which altogether contribute to silencing at telomeres and play a role in the regulation of telomere length [17,48] (Figure 1B). Binding of the SIR-complex to telomeres results in downregulation of transcription of subtelomeric genes—the effect known as Telomere Position Effect (TPE) [17,84]. Sir2 is a histone-deacetylating enzyme which promotes chromatin compaction mostly through deacetylation of the H4 histone at the K16 residue. Consistently, the subtelomeric yeast DNA which is characterized by the nucleosomal organization has decreased acetylation on H4K16 and possesses heterochromatic properties [17,48].

Remarkably, both human and yeast telomeric repeat sequences were found to disfavor nucleosome assembly and alter nucleosome positioning when studied in the yeast minichromosome system [92]. This effect can be mediated by the properties of the telomeric DNA repeats themselves and/or can result from tight protein binding to those repeats. In yeast, Rap1 as well as Abf1, Reb1, and Tbf1 belong to the General Regulatory Factor (GRF) DNA-binding protein group, the members of which regulate many aspects of DNA metabolism and bind to multiple sites in the genome. Numerous studies implicate Rap1, Abf1 and Reb1 in nucleosome exclusion at promoters and other sites [93,94,95,96,97,98,99,100,101,102,103]. The TTAGGG-binding factor Tbf1 was also found to contribute to nucleosome exclusion at some promoter regions and around DSBs and may facilitate NFR formation or affect positioning of nucleosomes [90,94,100,104]. Interestingly, DNA regions deprived of nucleosomes, i.e., Nucleosome Free Regions (NFR), or regions with irregularly spaced nucleosomes could provide a barrier against heterochromatin spreading and act as insulators subdividing genome into functional domains [105,106,107,108]. Direction of genomic loci to nuclear subcompartments was also proposed as mechanism accounting for insulation by GRFs [109].

In higher eukaryotes, telomeric chromatin is usually organized in arrays of nucleosomes spaced by short linker DNAs. However, unusual bipartite chromatin structure was also reported for human telomeres [91,110]. Telomeres and subtelomeres in mammalian cells harbor specific histone posttranslational modifications (PTMs). They include trimethylation at H3K9 and H4K20 along with hypoacetylated histones H3 and H4 and enrichment in heterochromatin protein 1 (HP1) [111]. These are all signs of constitutive heterochromatin. However a recent study shows that human telomeres have a lower level of H3K9me3 compared to heterochromatic Satellites II and III and are enriched in some euchromatic marks such as H4K20me1 and H3K27ac marks [112]. This observation is in agreement with other studies showing that telomeres carry less heterochromatic marks than subtelomeres [113,114,115]. *Arabidopsis thaliana* telomeres were also reported to exhibit euchromatic features while subtelomeres are organized in well-defined heterochromatic domains [116]. 

Telomeric DNA poses difficulties for the replication machinery owing to its repetitive nature, its ability to form alternative secondary structures, as well as to the tight binding of telomere-specific proteins [40,117,118,119,120,121,122,123,124]. The unwinding of G4-structures or dismantling T-loops or DNA/RNA hybrids requires specialized enzymes capable of dealing with these structures. Thus, various specialized DNA helicases are essential for telomere replication. They include helicases of the Pif1 family (Pif1 and Rrm3), RecQ-like DNA helicases (BLM, WRN, RECQL4, and yeast enzymes Sgs1 and Hrq1), Fe-S helicases (RTEL1 and FANCJ), UvrD/Rep type helicases (yeast Srs2 helicase), FANCM family helicases (FANCM and its yeast counterpart Mph1), and SWI/SNF-related helicases (HLTF, SHPRH, SMARCAL1 and yeast Rad5) [125,126,127,128,129,130,131]. 

Replication of yeast telomeres is usually initiated late in the cell cycle and replication forks move slowly through telomeres compared to other regions in the genome [17]. At the same time, short yeast telomeres are replicated in the early S-phase [132]. The replication timing of yeast telomeres is regulated by the Rif1 protein [133,134,135,136,137,138]. The pattern of human telomere replication is different. By-and-large, telomeres in human cells are replicated in mid S-phase, except for those in close vicinity to satellite sequences or localized at nuclear periphery [139]. 

Telomeres are also transcriptionally active. TERRA is a noncoding RNA consisting of telomeric repeats transcribed from the C-rich strand of a telomeric DNA repeat [140,141,142]. TERRA transcripts have been detected in all studied eukaryotic organisms from yeast to human and this RNA is now generally acknowledged as one of the key regulators of telomere homeostasis and length. Similar to telomeric DNA, TERRA can form G-quadruplexes in vitro, stabilized by Na+ or K+ cations, which renders it RNase resistant [143,144,145]. TERRA may also form stable RNA-DNA hybrids with the C-rich strand of a telomeric repeat, making the displaced G-DNA strand prone to G-quadruplex formation [146,147]. The resultant R-loop may have a tremendous impact on telomere stability. R-loops were proposed to impair replication fork progression, cause formation of DNA breaks and mediate HR-dependent telomere elongation [146,148,149,150]. It was speculated, therefore, that R-loop mediated recombination may regulate telomere maintenance in tumor cells by utilizing a mechanism of Alternative Lengthening of Telomere (ALT) (see below) [151,152]. TERRA abundance significantly increases at short and very short telomeres in yeast which promotes targeted homology-directed repair (HDR) [153,154,155]. TERRA is specifically associated with heterochromatic marks such as heterochromatic protein HP1 and histone H3 modification H3K9me3 and has been proposed to mediate formation of heterochromatin [149,156,157,158]. Due to base complementarity, TERRA can form a complex with telomerase RNA and initial studies suggested that TERRA can inhibit telomerase activity [159,160]. Subsequent studies revealed that TERRA acts instead as a positive regulator of telomere length [149,153,161,162].

Interestingly, RNA molecules complimentary to TERRA are also present in the cells. С-rich RNA transcripts synthesized from telomeres (ARIA) and subtelomeres (ARRET, αARRET) were described in fission yeast and plants [157,163,164,165]. ARRET transcribed from subtelomeric Y’ elements was also detected in budding yeast [159]. It is speculated that TERRA and ARIA may base pair in vivo with the formation of siRNA [164].

### 1.4. Regulation of Telomere Length: Alternative Mechanisms

Multiple facts point to telomeres as key regulators of cell proliferation potential, consequently their influence on lifespan control is intensively discussed [21]. Telomeres gradually shorten in somatic cells with aging [21]. The proliferative potential of pluripotent stem cells (PSC) and their genome stability also depends on telomere maintenance pathways [166,167]. Finally, nearly all hereditary human diseases associated with preliminary aging are somehow interrelated with changes in telomere length regulation or capping [21].

**Figure 2:**
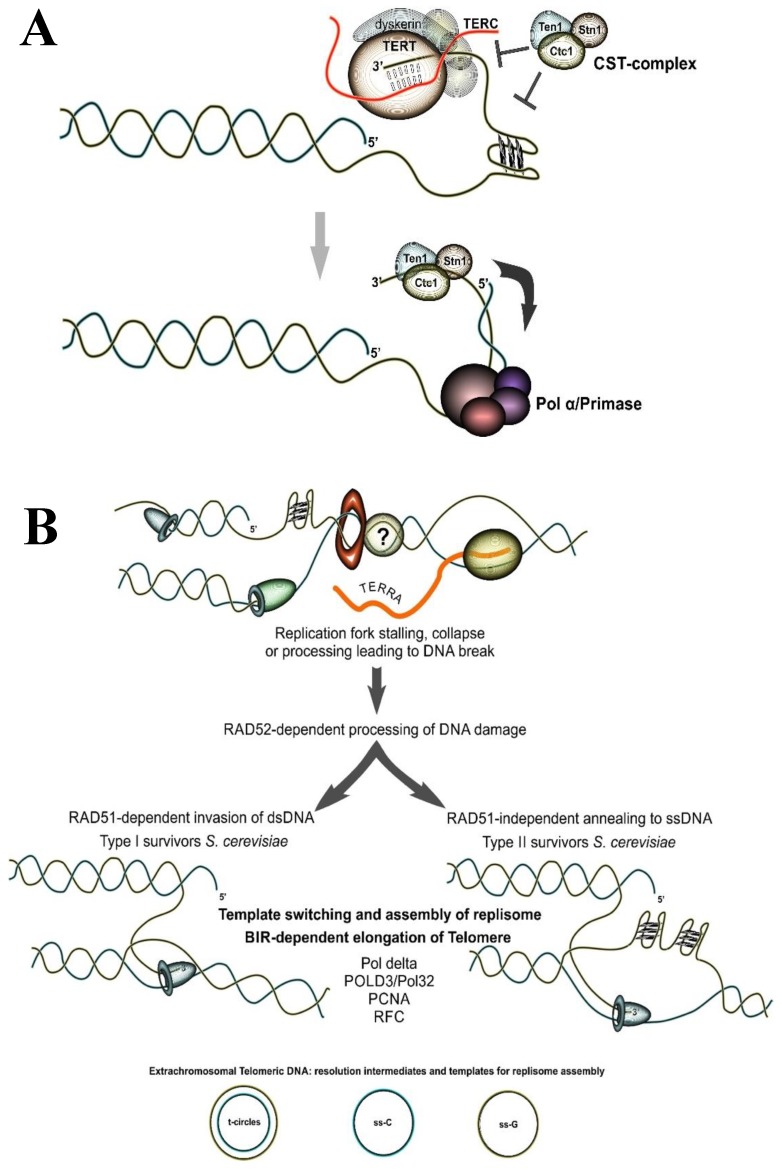
Telomere length maintenance mechanisms. (**A**) A simplified view of telomere length control by telomerase and the CST-complex. Telomerase adds telomeric repeats to the 3’-end of the G-rich overhang. Its activity is controlled by the CST-complex, which displaces telomerase, removes secondary structures and recruits the DNA polymerase α/primase complex to synthesize the C-rich strand. Note that regulation of telomerase by the CST-complex is more complex in yeast *Saccharomyces cerevisiae* (see text for details). (**B**) A general representation of the Alternative Lengthening of Telomeres (ALT) mechanism. ALT is currently viewed as the BIR-dependent elongation of telomeres after the replication fork stalling, ultimately leading to the formation of a one-ended double-strand break. This fork stalling can be due to the formation of unusual secondary structures in telomeric DNA (such as G-quadruplexes), the presence of stable DNA-RNA structures (R-loops generated during transcription of telomere-specific RNA called TERRA) or potent protein barriers (a circle with question mark). The replicative helicase is pictured as a red ring, RNA polymerase is a yellow oval and DNA polymerases are green and blue torpedoes with rings on their rear ends representing PCNA. One-ended DSBs are then channeled into the RAD52-dependent DNA damage repair pathway. This pathway is either RAD51-dependent, which involves invasion into a homologous or homeologous duplex, or RAD51-independent, which involves single-strand annealing. (These two pathways lead to the formation of Type I and Type II survivors in telomerase-deficient S. cerevisiae, respectively.) Upon template switching, the replisome is assembled and BIR-dependent synthesis of the telomere proceeds. BIR at telomeres requires DNA polymerase δ, its accessory subunit POLD3 (Pol32 in *S. cerevisiae*) as well as Replication Factor C (RFC) and PCNA (see text for details). The source of templates for RAD51-independent annealing is not limited to single-stranded regions released after G-quadruplex formation in the complimentary strand. For a comprehensive review of all eligible substrates see [50]. Importantly, such templates may be represented by extrachromosomal telomeric circular DNAs believed to be generated from the resolution of recombination intermediates at telomeres. Other sources for template DNA during BIR-dependent telomere-lengthening could be T-loops or interstitial telomeric sequences (ITSs) (for further discussion of these issues see Section 2.4).

The steady decrease of telomere length is caused by the semiconservative nature of DNA replication which leaves one of the strands, the synthesized lagging strand, incomplete in each cell cycle [5,6]. This process is complemented by the C-strand degradation required to generate the G-rich single-stranded overhang of the telomere. Actively dividing cells, including most cancer cells, address telomere shortening with the help of the telomerase complex, whose reverse transcriptase adds telomeric repeats to the G-rich chromosome end [7,152,168]. After that the DNA polymerase α /primase is recruited to synthesize the complimentary C-strand [169,170,171,172,173,174,175]. The CST complex is a major regulator of telomere DNA synthesis by regulating the activities of both telomerase and DNA polymerase α /primase complexes [59,60,64,176,177,178,179,180,181,182,183,184] (Figure 2A). In yeast, the role of CST is more complex: it additionally involves telomere capping [169,170,185,186,187,188,189,190]. While many aspects of the regulation of telomerase activity have been extensively studied, several details of this process in yeast and in mammals remain to be elucidated. One perplexing question is what mechanisms are responsible for species-specific telomere length homeostasis? Current models include the protein-counting mechanism and/or the replication fork model [191,192]. The first model implies that telomere-bound proteins can block telomerase’s access to the termini at a distance. Consequently, longer telomeres would inhibit telomerase more strongly than the shorter ones owing to the larger number of “telomerase repressors.” The second model postulates that telomerase is delivered to the telomere termini by the replication fork, thus, telomere length homeostasis might depend on the efficiency of fork progression through telomeric chromatin and telomere-bound proteins. While further studies are needed to elucidate the fine balance between telomere shortening and lengthening inside dividing cells, the second model is in-line with our data, which show that replication fork stalling at cloned telomeric repeats increases with their length and depends on the presence of the Rap1 protein [123].

Comprehensive research suggests that telomere length can be maintained in dividing cells in the absence of telomerase, reviewed in [193,194]. Alternative lengthening of telomeres (ALT) is a telomere maintenance mechanism (TMM) in the absence of telomerase [8,195,196] (see Figure 2B). In humans, ALT accounts for cell immortalization in 10–15% of cancers. A high percentage of ALT occurrence is typical for tumors arising from bone (62%), soft tissue (32%), neuroendocrine systems (40%), peripheral nervous system (23%), and the central nervous system (15%) [152]. Utilization of ALT by a tumor portends a poor prognosis in the majority of cases. While ALT was initially described in telomerase-negative tumors, it is becoming progressively more evident that it might co-exist with telomerase-based telomere maintenance [197]. For example, ALT was linked to early stages of leukemogenesis in chronic myeloid leukemia, where it enhances the proliferative advantage of the tumor cell population that is then maintained through the activation of telomerase [198]. ALT traits were also detected in normal somatic cells in mice and non-neoplastic cells in human [199,200]. Furthermore, telomeres can lengthen significantly through an ALT-like recombination-based mechanism during early development in mice and in plants [201,202]. 

ALT is a widespread phenomenon—it exists in lower and higher eukaryotes. Shortening of telomeres in telomerase-deficient (*est1*, *est2* or *tlc1*) yeast strains causes cellular growth senescence [203,204,205]. This senescence can be overcome via a *RAD52*-dependent bypass pathway responsible for the generation of “survivors”, which are able to maintain telomeres in a telomerase-deficient background [8,206,207,208,209,210,211] (see Figure 2B). Further analysis of these *RAD52*-dependent survivors revealed two groups: the *RAD51*-dependent survivors (Type I) and the *RAD51*-independent *RAD50*-dependent survivors (Type II) [207,208]. Type I survivors were more common (approx. 90%) but can eventually convert into stable Type II survivors [206,212]. 

Type I survivors maintained their ends by tandem amplification of subtelomeric Y’ elements and were dependent on *RAD51, RAD54, RAD57*, and *POL32* genes [8,208,211]. Type II survivors maintained telomeres by elongating the distal C_1–3_A/TG_1–3_ telomere repeats and they often bear very long (ranging up to 12kb) and highly variable-length tracts of C_1–3_A/TG_1–3_ repeats [206]. Type II survivors occur in a *RAD59*-dependent pathway which requires the MRX protein complex as well as Sgs1 and RecQ helicases and Tel1 and Mec1 checkpoint kinases [208,209,210,213,214]. Unexpectedly, deletion of either *EXO1* or *SGS1* abrogated the inability of the *tlc1 rad52* mutant strains to generate survivors [215,216]. The clones appearing in *tlc1 rad52 exo1* background were called PAL-survivors; they lacked telomeres and were proposed to use acquired palindromic sequences to form covalently closed hairpins to cap their chromosomal ends [215]. Survivors selected in the *tlc1 rad52 sgs1* background demonstrated similar features and accumulated an increased level of chromosomal Ty1 sequences [216]. 

Several lines of evidence suggest that DNA polymerase δ mediated Break-Induced Replication (BIR) is responsible for generation of both Type I and Type II survivors (see Figure 2B). Yeast strains defective in the catalytic activity of the Pol δ were not able to amplify the Y’ elements nor could they support significant elongation of the terminal C_1–3_A/TG_1–3_ repeats [214]. In addition, the *POL32* gene, encoding the accessory subunit of the DNA polymerase δ, is absolutely paramount for generation of both types of survivors [211]. The double *tlc1 pol32* mutant was not able to generate survivors similar to the *tlc1 rad52* or the *tlc1 rad51 rad59* mutant strains [208,211]. Other studies suggested that generation of the Type I survivors depends mostly on the activities of the DNA polymerase ε, the DNA polymerase α and the Cdc13 protein [214,217]. 

ALT human cells typically have long, very heterogeneous telomeres that fluctuate in length and in most cases resemble the Type II survivors in yeast. In addition to the high level of sister-chromatid exchange and extrachromosomal telomeric DNA, ALT cells also accumulate specific subnuclear structures—ALT-associated promyelocytic leukemia (PML) nuclear bodies (APBs), which consist of telomeric DNA and proteins [195,218,219,220,221,222]. Telomere extension in ALT-positive tumor cells can be efficiently induced by DSB formation and appears to occur as a conservative DNA replication process similar to Break-Induced replication (BIR), requiring Pol δ, PCNA, RFC as well as the accessory subunits of Pol δ, the POLD3 the homolog of yeast Pol32, and POLD4 [223,224,225,226] (see Figure 2B). Variant repeats, such as TCAGGG repeats are often found at ALT telomeres and their appearance is attributed to Pol η activity, which regulates the ALT mechanism by alleviating the replication stress [226,227,228]. 

Recent studies described an altered chromatin state in ALT human cells. ALT cancer incidence correlates with mutations in the ATRX/DAXX chromatin remodeling complex and histone variant H3.3 [229,230,231,232]. Depletion of histone-chaperone ASF1, which facilitates histone deposition and exchange during nucleosome assembly, resulted in the induction of ALT activity in telomerase-positive cells [233]. Chromatin compaction is reduced at ALT telomeres and is associated with reduced H3K9 trimethylation and upregulation of telomere transcription [234]. Variant TCAGGG repeats, which intersperse canonical telomeric repeats at ALT telomeres, are binding sites for orphan nuclear receptors of the NR2C/F classes which promotes spatial proximity of telomeres and telomere-telomere recombination [235,236,237]. These receptors are also able to recruit the NuRD histone deacetylation complex which induces Shelterin removal, chromatin compaction and consequent replication stress [238]. NR2C/F facilitate tethering of telomeric chromatin to thousands of non-telomeric sites throughout the genome and mediate genome rearrangements and targeted telomere insertion in ALT cells [237]. As it was noted, telomeres are transcriptionally active and TERRA RNA is considered as one of the key regulators of telomere length. R-loops formed with TERRA are thought to mediate DNA recombination and DNA repair synthesis in tumor ALT cells [151,152,225]. TERRA specifically contributes to the Type II survivors in yeast and the abundance of this RNA is controlled by several factors including the Rat1 and Rnh201 nucleases, the Sir2/Sir3/Sir4-complex, proteins Rif1 and Rif2 and the THO-complex which promotes co-transcriptional RNA export [159,239,240]. Short telomeres fail to recruit a sufficient amount of Rat1 and Rnh201 nucleases which results in TERRA accumulation, impeding replisome progression and activating HDR [155,159]. These and other studies suggest that ALT-mediated telomere extension depends on chromatin state and on the interplay of replication and transcription machinery with unusual chromatin and nucleic acid structures at telomeres.

## 2. Interstitial Telomeric Sequences (ITSs)

### 2.1. ITSs and their Proposed Origin

Telomeric repeats are not found exclusively at the ends of chromosomes. In fact, telomeric repeats are present in multiple internal sites of chromosomes in many species. Such sequences are called Interstitial Telomeric Sequences (ITSs). They are found in most vertebrates including human and in some plants [14,241,242,243,244,245,246,247,248,249,250,251,252,253,254]. Based on cytogenetic analysis in vertebrates, ITSs are divided into two major groups: heterochromatic ITSs (het-ITSs) and short ITSs (s-ITSs). Het-ITSs are large blocks of telomeric repeats that are present mainly in centromeric or pericentromeric regions. In contrast, short ITSs (s-ITSs), stretches of a limited number of TTAGGG hexamers, are distributed at various positions in chromosomes [246]. There are also blocks of degenerate (TTAGGG)-like repeats in subtelomeric regions (subtelomeric ITSs), which together with the adjacent ARS, divide subtelomeres into proximal and distal areas, - an arrangement conserved between yeast and humans [255,256]. In some species, such as the Chinese hamster, het-ITSs constitute the major component of satellite DNA sequences and they co-localize with sites of chromosomal breakage. These large blocks of telomere-like repeats are often viewed as remnant scars of gross chromosomal rearrangements that occurred during karyotype evolution [244]. The human genome lacks very long het-ITSs; some human ITSs seem to reflect head-to-head blocks of telomeric repeats derived from terminal fusion of ancestor chromosomes that gave rise to modern human chromosomes [244,253,257,258]. For example, the 2q13-2q14 ITS on human chromosome 2 and 1q41 ITS on human chromosome 1 are classified as fusion ITSs [246,253]. End-to-end joining of two telomeres such as in case of Robertsonian fusions are frequent events in the evolution of vertebrate karyotypes that can result in het-ITS formation [246,259,260]. The ability of ITSs to undergo massive amplification can lead to the formation of extra long megabase-sized ITS arrays in centromeric or pericentromeric areas which in some cases are longer than genuine telomeres [244,245,246,261,262]. The mechanisms proposed to explain amplification of ITSs include unequal crossing-over, gene conversion, DNA replication slippage and rolling circle replication of extrachromosomal circular DNA (ecDNA) [246]. Other possible mechanisms of het-ITS formation could be transposition of telomeric repeats by mobile elements or translocation of an ITS in the course of genetic recombination. Supporting the latter ideas, ITSs are often flanked by transposable mobile elements and satellite DNA [246]. 

Most interstitial telomeric sequences studied in the human genome are short (s-ITSs) with lengths varying from 2–25 copies [244,247,251,253,263]. They are present in all human chromosomes in subtelomeric regions as well as far from chromosomal ends [246,264]. Positions of some s-ITSs are conserved between human and other primates, while others appear to have arisen late in primate evolution and thus are only present in humans and chimpanzees [242,243,265]. Remarkably, recent high-throughput approaches have revealed that the total count of various ITSs in the human genome reaches several thousands. At least 714 ITSs were listed in USCS Genome Browser [266], and 2920 ITSs were reported in [267]. As suggested by phylogenetic analysis, most s-ITSs resulted from insertions of telomeric repeats when a double-stranded break in DNA was repaired by non-homologous end joining with possible telomerase recruitment [263,265]. Recent studies have supported this idea. Insertion of telomeric sequences de novo at etoposide-induced DSBs occurs in ATM/ATR- and telomerase-dependent mode [268,269]. Moreover a targeted telomere insertion (TTI) mechanism mediated by the NR2C/F orphan nuclear receptors and promoting jumping of telomeric sequences to hundreds of sites in the genome was recently proposed for human ALT cells [237] (see also Section 1.4). As it was discussed in chapter 1.4, NR2C/F nuclear receptors bind to TCAGGG repeats that intersperse ALT telomeres and to hundreds of regular NR2C/F-binding sites throughout the genome [236,237]. Bringing together telomeric chromatin and endogenous non-telomeric NR2C/F binding sites may promote their recombination, resulting in genome infiltration by telomeric repeats.

In some species, the origin and evolution of ITSs remains puzzling. Examples of mixed telomeres consisting of different types of telomeric repeats along with the presence of different types of ITSs in the same genome were described in plants [270,271]. Some plants, such as in *Cestrum elegans*, contain diverged repeats in both ITSs and telomeres: their ITSs are composed of (TTTAGGG)n repeats while telomeres are made up of (TTTTTTAGGG)n repeats [272,273]. Another interesting example is yeast *S. cerevisiae*, which has TG(_1–3)_ telomeres and low copy arrays of human-type TTAGGG repeats as well as degenerative TG_(1–3)_ repeats at subtelomeres. It is hypothesized that a yeast ancestor had the TTAGGG motif at telomeres that was later substituted by the TG_1-3_ degenerative motif due to mutation of the telomerase RNA template (see also Section 1.3.2). 

### 2.2. Genetic Instabilities Associated with ITSs

**Figure 3:**
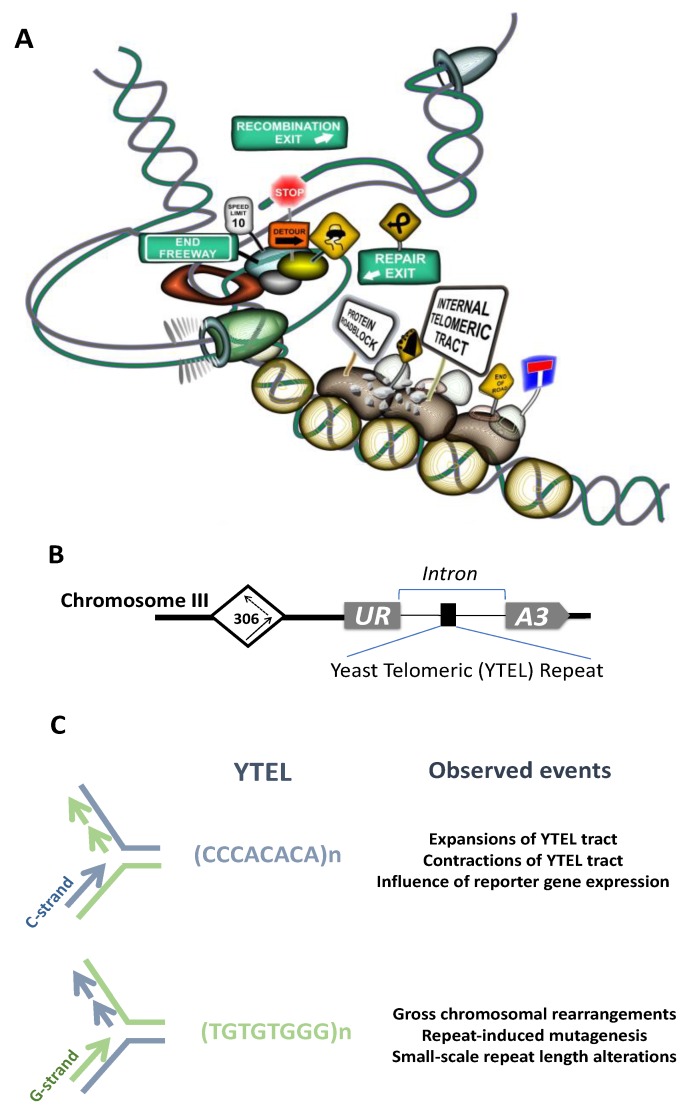
Mechanisms of Interstitial Telomeric Sequences (ITSs) instability (based on yeast model). (**A**) ITSs represent polar blocks for replication machinery [123,299,300]. Factors binding specifically to ITSs sequences (such as Rap1 in yeast, yellow stones on the figure, “protein roadblock” sign) influence the replication passage through this sequence depending on the orientation of the ITS tract. Additional factors interacting with Rap1, such as Sir-complex or Rif1 and Rif2 proteins (brown and gray stones), may enhance the stalling of the replication fork and regulate the replication, transcription and repair processes at ITS. Replication stalling is facilitated by the replication fork pausing complex component (Tof1-Csm3 in yeast, “stop,” “end freeway” signs) and can lead to single-stranded gap (ss-gap) formation. The stalled replication forks and ss-gaps can be repaired via HR (“recombination exit” sign) or postreplication DNA repair (“repair exit” sign). Template Switching (TS) (“detour” sign) is essential for both pathways. Replication from the other direction can result in replication fork collapse and DSB formation (“end of road” sign). In this case the gross chromosomal rearrangements can be formed. (**B**) The yeast model system we used to study ITS. The *URA3* reporter gene, which is commonly used for both direct and counter-selection, is split with an artificial intron carrying the insertion of the telomeric tract. The UR-Int(YTEL)-A3 reporter cassette is placed in the chromosome III near ARS306. Placing telomeric tracts of varying length in different orientations and selection for the reporter gene inactivation events (on 5-FOA media) or reporter gene activation events (Ura- media) allows us to select for different events induced by the ITS tract as well as the length of the ITS tract. (**C**) The overview of the events induced by the ITS tract depending on its orientation. G- and C-rich strand denote the lagging strand template for replication and sense strand for transcription.

Cytogenetic analyses implicated ITSs in spontaneous and induced chromosome breakage and rearrangements in primates [242,243,274,275], rodents [276,277,278,279,280], fish [281,282,283], plants [271,284] and human [285]. Large blocks of ITSs such as het-ITSs observed in some species are supposed to confer even more fragility and contribute to genome evolution [244,286,287,288]. Closely related species with similar chromosome morphology can reveal different patterns of ITSs distribution, reflecting the dynamic nature of ITSs [262,289]. Similarly to other microsatellites, ITSs show substantial length polymorphism [252,264,290,291,292] and can undergo spontaneous amplification in CHO cell lines [246]. Moreover, some genotoxic compounds were shown to induce ITSs amplification and accumulation [246]. Hypervariability of ITSs within a single species was recently reported in plants [284]. Telomeric repeats are highly recombinogenic in yeast [293], rodents [294,295,296] and humans [297]. These data pointed to the idea that ITSs could represent a source of significant genetic instability. Additional confirmation of this idea came from experiments where an 800 bp-long telomeric tract was inserted into an intron of the APRT gene, which induced deletions and rearrangements of the reporter APRT gene in CHO cells [298]. Data in recent years presented direct evidence of ITSs being a source of chromosomal fragility, rearrangements etc. Thus, the ITS at 2q14 on human chromosome 2 behaves as a common fragile site and requires the Shelterin component TRF1 for its stabilization [285]. Our experiments in yeast proved direct involvement of the interstitial telomeric tract in induction of various types of chromosomal rearrangements [299]. Short tracts of yeast telomeric (Ytel) repeats placed at an internal chromosome position in an orientation such that G-rich strand served as the lagging-strand template during replication stimulated gross chromosomal rearrangements and repeat-induced mutagenesis (RIM) [299]. In the opposite, C-rich orientation, ITS demonstrated high rates of repeat expansions and contractions [300] (see Figure 3A–C for an overview of the system, events induced by the ITS tract and a graphic illustration of the mechanisms responsible for these instabilities).

These observations could be quite relevant to what is observed in human disease. S-ITSs show elevated length polymorphism in some tumors [252,290]. ITS sites were implicated in the formation of so-called jumping translocations where a fragment from a donor chromosome is transferred to several recipient chromosomes [301]. For instance, ITSs were often found at translocation junctions in patients with Prader-Willi syndrome [302,303,304,305,306,307], Dandy-Walker malformation [308] and hematopoietic malignancies including acute myeloid leukemia (AML) [309,310,311]. Several reports show involvement of ITSs in constitutional chromosomal abnormalities [307,312,313,314]. ITSs were also found at breakpoints of unbalanced translocations observed in neuroblastoma [315]. It is believed that somatic recombination between ITSs and telomeres may underlie some of these phenomena, though more studies clearly should be done to understand mechanisms of ITSs instability. Interestingly, ITSs accumulation in various genomic regions seems to be associated with karyotypic changes in cancer development. ITSs accumulate in ALT cancer cells and the TTI mechanism is thought to contribute to the complex karyotypes found in such tumor cells [237]. Cells heterozygous for BRCA2 mutations associated with breast cancer also accumulate an extensive amount of ITSs, which can be explained by elevated formation of DNA breaks during replication and their subsequent healing involving telomerase [316].

### 2.3. Factors Binding to ITSs and Proposed Functions of ITSs in the Genome

In yeast, we and others found Rap1 binds to an artificial interstitial telomeric tract, which was suggested to cause an orientation-dependent protein roadblock during replication, followed by DSB formation and their consequent repair associated with formation of chromosomal rearrangements [123,299,317] (see Figure 3A). Another factor with confirmed binding to ITSs sequences in yeast is the yKu heterodimer, which binds at subtelomeric ITSs and an artificial ITS site in the yeast genome [318]. The yKu heterodimer, consisting of Yku70 and Yku80 proteins, is a major player of NHEJ helping to repair DSB breaks by sealing non-homologous ends together. It was proposed that binding of yKu to such sites in the genome might be provoked by replication fork stalling at ITSs sites followed by fork reversal or DSB formation [318]. Interstitial tracts of TTAGGG repeats that are also present in yeast subtelomeric regions (see above) are bound by the Tbf1 protein and this interaction plays an essential role in stability of these sequences (Aksenova and Radchenko, preliminary data).

There is mounting evidence that Shelterin components occupy selective interstitial telomeric sites in the human genome. RAP1 and TRF2 occupy a fraction of ITSs where they are thought to participate in the regulation of transcription [319]. Another study revealed binding of TRF1 and TRF2 to 48 intrachromosomal regions including a subset of 30 different short ITSs and the 2q14 fusion ITS in a cancer cell line [266]. This binding of TRF-proteins has a preference for genic regions and depends significantly on sequence conservation and the length of ITSs [266]. Long artificial 800 bp-long ITSs showed enrichment in TRF1 and TRF2 proteins, as well as in the TRF2-interacting partner, Apollo exonuclease [266,320]. TRF2 and TIN2 co-localized with an ITS derived from the translocation event in a patient with a rare chromosomal abnormality [321]. The fusion region 2q14, containing stretches of degenerate TTAGGG repeats, binds TRF1, TRF2, RAP1, and TIN2 proteins [258,285]. Notably, reduction of TRF1 binding to the 2q14 region increased the frequency of aphidicolin-induced breaks within this region whereas repression of TRF2 expression did not. Targeting of Bloom’s syndrome helicase (BLM) and ATR kinase by shRNA also affected the stability of the 2q14 region [285]. Co-localization of Shelterin components with ITSs was also reported for other species. TRF1 was shown to bind large interstitial telomeric blocks in chinese hamster ovary cells, protecting these sites from breakage and rearrangements [322,323]. RAP1 in a complex with TRF2 was found at interstitial telomeric and subtelomeric repeats in mice, where it appears to regulate transcription [77]. In a murine model, deletion of mPOT1 promoted development of invasive breast cancers accompanied with p53 inactivation. Interestingly, massive telomeric amplification and formation of very long ITSs encompassing in some cases entire chromosomes were observed in such tumors [324]. A recent study shows that ubiquitin C-terminal hydrolase isozyme L1 (UCHL1), a protein which is strongly expressed in neurons, binds to ITSs including unstable ITSs in the regions 2q31, 21q22 and common fragile ITS site at 2q14. UCHL1 is able to interact with several Shelterin proteins and the role for UCHL1 might include modulation of the interaction between Shelterin-bound telomere or ITSs and the nuclear scaffold [325]. Given that UCHL1 expression is affected in many non-neuronal tumors its binding to ITSs might be relevant for the disease onset or progression.

**Figure 4:**
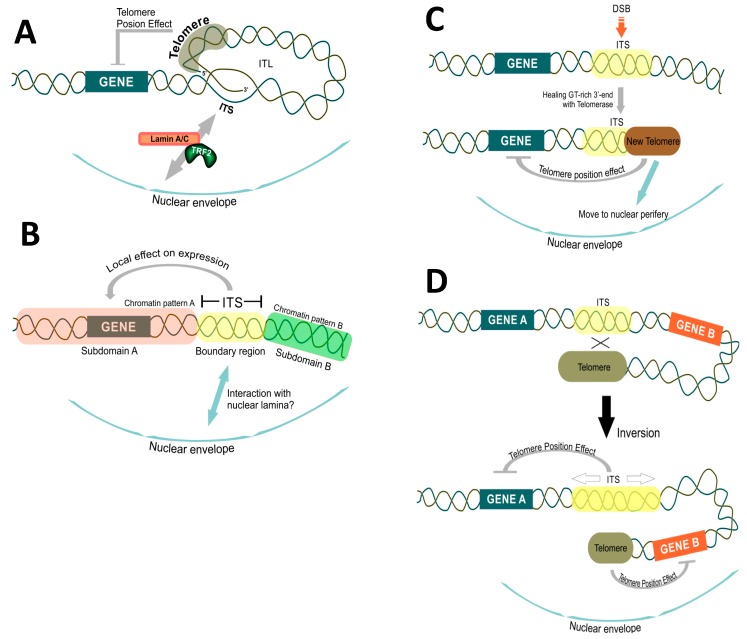
Possible roles of ITSs in the genome. (**A**) ITSs can participate in the formation of the Interstitial t-loop (ITL). This ITL can play an essential role in telomere maintenance and regulation of gene expression. Mediated by Lamin A/C and TRF2, the interaction of the ITS with the telomere can regulate the chromosome structure and position into the nucleus. Juxtaposion of the telomere and ITS may influence expression of genes near the ITS [267,297,326,329,330]. (**B**) ITSs can serve as boundary elements in the genome. Through recruitment of proteins that specifically bind to telomeric repeats and possess general regulatory activity, such as Rap1 in yeast [109] (yellow oval), ITSs can regulate the structure of adjacent chromatin and divide the chromosome into subdomains (orange and green rectangles). A possible interaction of ITSs with the nuclear lamina may regulate the 3D genome structure. (**C**) ITSs are potent elements conferring karyotype plasticity. ITSs can serve as spare sites for telomere formation and can impact the outcome of DSB repair [335] and can seed a new telomere. (**D**) Recombination between the ITS tract and the telomere can result in the inversion of the chromosome arm with a consequent effect on gene expression [299,336].

Recent studies point to an essential function played by ITSs in the stability of the genome and specifically at the role played by ITSs in interacting with the nuclear envelope and shaping the genome’s 3D structure (see Figure 4A). A novel model of mammalian chromosomal organization that involves interaction of telomeres with ITSs and nuclear lamins was proposed in [267,297]. It resulted from the observations that T-loops can be formed at interstitial telomeric sequences (interstitial telomeric loops, ITL) and that they are stabilized by TRF2 binding and the TRF2-interacting lamin A/C. Lamin A/C is a crucial component of the inner nuclear membrane envelope and is encoded by the LMNA gene. The well-known mutation in this gene leads to synthesis of a permanently farnesylated form of protein, called progerin. Progerin accumulates in people suffering from a premature ageing disorder called Hutchinson Gilford progeria syndrome (HGPS) which also results in telomere shortening and decreases lifespan. Strikingly, progerin is unable to interact with TRF2, which may reduce the formation of ITLs resulting in telomere instability [267,297]. The formation of loops between telomeres and distant chromosomal was also suggested in other studies, and these structures were implicated in the regulation of gene expression at a distance [45,46,326,327,328]. In some cases, this phenomenon may affect genes located up to 10 Mb away from the telomere, hence it was termed Telomere Position Effect Over Long Distances (TPE-OLD) [326]. Interaction between ITS at the hTERT gene, hTERT promoter and subtelomeric 5p region mediated by TRF2 is responsible for TPE-OLD: bringing together these regions made a loop that influenced hTERT expression [329,330]. Remarkably, analysis of TRF-binding sites in the human genome revealed that they are enriched in the proximity of genes or within introns, implying that Shelterin proteins may couple telomeres and ITSs, controlling genome architecture and gene regulatory network. The balance in the amount of TRF2 bound to telomeres versus non-telomeric sites might, thus, affect hTERT transcription and telomere length regulation [331]. 

ITSs may subdivide the chromosome structure and influence the chromatin organization in the adjacent regions thus serving as genome partitioning elements. As it was discussed above, ITSs in lower and higher eukaryotes are bound by specific protein factors and both yeast and human telomeric repeats disfavor nucleosome assembly (see Section 1.3.3). Moreover studies in yeast showed that proteins binding to telomeric repeats, such as Rap1 and Tbf1 (binding to TTAGGG repeats), may act as nucleosome exclusion factors. Rap1 and Tbf1 are endowed with potent insulator capacity and are proposed to act as genome partition elements [109,332]. Yeast and human telomeric repeats in our model system also affected the reporter gene expression [299,300] (Aksenova and Radchenko, preliminary data). We speculate, therefore, that ITSs may function as elements which subdivide the chromosome into functionally independent regions (Figure 4B).

Another important observation is that ITSs can be transcribed. Both TERRA and ARRET transcripts synthesized from interstitial telomeric positions were found in *Bonnaya antipoda* and *A. thaliana*, with their transcription peaking in blossom [163,165]. TERRA and ARRET transcripts in *A. thaliana* formed partially double-stranded intermediates processed by Dicer-like activity into heterochromatic small interfering RNA (siRNA). Such siRNAs were found in association with Argonaut 4 and were apparently involved in the RNA-dependent DNA methylation (RdDM) of cytosine residues in asymmetric sequence context (CNN methylation) [163]. RdDM is a versatile epigenetic tool in plants allowing dynamic regulation of gene expression which promotes physiological flexibility and adaptation to the environment [333,334]. ITSs in *A. thaliana* demonstrate clear heterochromatic features, such as enrichment in H3K9me2, H3K27me and cytosine methylation [116]. Moreover, siRNA-driven heterochromatinization of ITSs was suggested to be a mechanism preventing ITS-mediated genome instability [116,163]. In vertebrates, large ITS blocks are usually observed within regions of constitutive heterochromatin [248] which implies that ITSs may be intrinsically prone to heterochromatinization through siRNA or other mechanisms. 

In some organisms, ITSs are localized to Nucleolus Organizer Regions (NOR) or interspersed with rDNA [248,281,289,337,338,339,340,341,342,343]. It was, thus, speculated that telomeric sequences and specifically ITSs might play a role in nucleolus organization [343]. TRF2 protein association with the nucleolus and rDNA is also of interest in this context [344]. Yet another hypothesis about ITS’s function in the genome assigns them a role in centromere repositioning [275,281,345,346] and telomere formation [248]. For instance, it was proposed that ITSs can serve as spare sites for telomere formation, thus increasing karyotype plasticity [248] (see Figure 4C). Supporting this idea, the presence of Rap1-bound to TG-rich sequence at a DSB blocked Mre11-Rad50-Xrs2 recruitment, impaired resection of the broken end and favored its elongation by telomerase [335]. In our yeast system, we observed the formation of an acentric minichromosome as a result of ITS-mediated chromosome rearrangements induced by ITS where the TG rich 3’-end originating from the yeast telomeric interstitial tract seeded new telomere [299]. Another mechanism adding genome plasticity through ITSs is formation of terminal inversion between artificial ITS and the left telomere on yeast chromosome III [299,336]. Our data suggest that ITSs do interact with telomeres and such interaction can result in karyotype changes, such as terminal inversions, affecting the position of genes in the chromosome and their expression (Figure 4D). Importantly, our data support the idea that complex karyotypic changes associated with ITSs and observed in human diseases (discussed in Section 2.2) do result from the interaction between ITSs and telomeres. 

Altogether these examples strongly imply that ITSs are important genomic elements rather than useless junk DNA. 

### 2.4. Mechanisms of ITS-Mediated Genome Instability

The data described above implicate ITSs as hotspots for DNA breakage and genome instability. The mechanisms responsible for this instability are not completely understood. We have recently set up a genetically tractable yeast experimental system to study the mechanisms of ITS-mediated genome instability. When yeast telomeric repeats (Ytel) were placed inside the third chromosome such that G-rich strand served as the lagging-strand template during replication, they induced Gross Chromosomal Rearrangements (GCRs) and mutagenesis at a distance [299] (Figure 3B,C). In this orientation, Ytel repeats are extremely potent inhibitors of DNA replication, which is caused by binding of the Rap1 protein [123,299,317]. In the opposite orientation, Ytel repeats caused a much weaker replication stall, and the major instabilities in this orientation were frequent repeat expansions and contractions [123,300] (Figure 3B,C). Tightly bound protein complexes can form a polar fork barrier and stall replication fork progression, which can ultimately lead to formation of single-stranded gaps behind the replication fork or a DSB [347]. Based on our data and data from other research groups, we favor a model that the orientation dependence of ITS-mediated genome instability stems from the asymmetry of protein-DNA complexes at telomeric repeats [118,123]. We suggest that binding of multiple Rap1 molecules to the interstitial Ytel repeat creates a polar block for the replication machinery, which can result in either fork collapse or a modest fork slowing, depending on the repeat’s orientation (see Figure 3A,C). The exact mechanisms responsible for the polar counter-replication activity of the Rap1 protein remains to be elucidated. It is also unclear whether Rap1-interacting factors such as Rif1 and Rif2 or Sir3 and Sir4 contribute to this polar effect, albeit data in [317] imply that Rif1, Rif2, Sir3, and Sir4 proteins are dispensable for Rap1-induced DSB formation during DNA replication. One possibility is that the Rap1 protein alone acts as a polar counterhelicase. On the other hand, Rap1 has the potential to reorganize chromatin and compete out nucleosomes [95,97,100,101,103,106,348,349]. Thus, another possibility is that a change in the local chromatin structure around the Rap1-bound telomeric repeats can impede progression of the replication machinery in an orientation-dependent manner. Finally, one can imagine that this orientation-dependence could be grounded in the differential structure-forming potential between the G-rich and C-rich strands of telomeric repeats. For example, a recent study of the *Pel* enhancer region instability in stickleback fish shows that (TG)_n_ repeats can promote mutagenesis in an orientation-dependent manner, correlating with formation of alternative secondary structures in the DNA [350]. Remarkably, the TG-rich orientation was found to be significantly more prone to DSB formation than the CA-orientation, which is similar to our observations.

We observed several types of GCRs mediated by the Ytel repeats [299]. They included inversion of the left arm of the chromosome III, gene conversion events between chromosomes III and V resulting in either deletion within chromosome III or deletions coalesced with duplications of the right arm of the chromosome III, and translocations between chromosome III and other yeast chromosomes. Gene conversion and translocation events were accompanied by the formation of an acentric minichromosome representing the fragment of the left arm of the yeast chromosome III. Break-induced replication (BIR) and homologous recombination (HR) are the mechanisms likely responsible for these genome rearrangements. 

Mechanisms of Ytel-induced terminal inversion were studied in detail [336]. This class of events was initiated by a DSB within the Ytel tract followed by the single-strand annealing (SSA) pathway leading to the annealing of the broken ITS on chromosome III to the left telomere of the same chromosome. Interestingly, this process involves the MRX complex, which likely acts by holding the ends of the inverted fragments together and the Rad1/Rad10 and Msh2/Msh3 factors, which are required to remove the single-stranded flap produced after annealing [336]. Given the data that MRX activity is inhibited when TG-rich sequences are proximal to the DSB [335], terminal inversions preferably occur upon DSB formation in the ITS. This mechanism might be of significance to confer karyotype plasticity. Another possibility could be the formation of an ITL-loop between the telomere and the ITS sequence [267]. Both phenomena imply direct interaction between an ITS and the actual telomere, which has major ramification for the genome stability and telomere length regulation (see also discussion in Section 2.3 and Figure 4C,D).

Repeat induced mutagenesis in the Ytel case was dependent on the activities of the MRX complex, Sae2 nuclease and the catalytic subunit of the DNA polymerase ζ, Rev3. We believe therefore, that it results from a post-replication gap-filling by the DNA polymerase ζ [336].

Importantly, in all studied cases, Ytel-mediated instability was provoked by the Tof1 and/or Csm3 proteins [300,336]. Tof1 and Csm3 are components of the replication-fork-pausing complex (RFPC) assisting replication fork protection and stabilization at natural impediments such as secondary DNA structures or protein blocks [351]. Tof1 and Csm3 enhance the replication fork stalling at tight DNA-protein barriers [352]. For instance, Tof1 promotes replication fork stalling at the Rap1-bound Ytel repeats, which ultimately leads to their instability [123,299,300,336]. TIMELESS and TIPIN proteins, which are homologs of the Tof1 and Csm3 proteins in mammals, are both important for replication at telomeres. TIMELESS interacts with the TRF1 and TRF2 proteins and its depletion slows telomere replication and leads to telomere shortening and disfunction [353]. Studies in fission yeast showed that depletion of TIMELESS homolog Swi1 induced telomere shortening which subsequently triggered amplification of telomeric DNA [354]. In ALT cancer cells, TIMELESS and TIPIN suppressed telomeric clustering and mitotic DNA synthesis at telomeres [225]. Importantly, multiple studies revealed that expression of TIMELESS and TIPIN is up- or downregulated in various cancers [355,356,357,358,359,360]. The association of RFPC components with ITS-mediated instability and ALT highlights the key role of replication stress and mechanisms leading to its alleviation in both processes.

**Figure 5:**
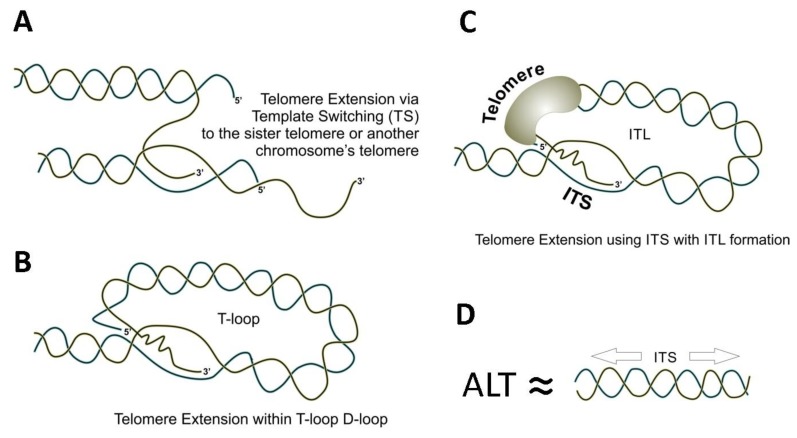
Alternative lengthening of telomeres and ITSs. (**A**) ALT is a process that relies on Template Switching (TS) and is thought to be initiated by replication blockage or a break in the DNA. The strand exchange reaction performed by specialized enzymes (e.g., TS helicases) or HR-proteins is followed by the DNA synthesis performed in BIR-like manner. The source for the template could be either the sister telomere or a telomere on another chromosome. (**B**) Extension mechanism via intratelomere invasion is also discussed [50,228]. (**C**) Another striking possibility is the usage of ITSs for telomere extension. In this case, an Interstitial t-loop (ITL) should be formed. The extension might occur via multiple cycles of invasion or DNA slippage. (**D**) Key components participating in ITS expansion are relevant for the ALT process [300], hence these two processes share certain similarities. An exciting prediction from this model is that ITS’s length destabilization can be an indicator of activated ALT.

It is becoming progressively clearer that ALT is a BIR-like process, although many aspects of this pathway of telomere elongation remain puzzling. BIR is defined as recombination-dependent conservative DNA replication, which involves template switching (TS) with invasion into the homologous duplex DNA followed by the formation of a migrating D-loop (“migrating bubble”) [50]. BIR is likely to be initiated by replication fork collapse at DNA-protein barriers within telomeres or ITSs (Figure 2B). Leading and lagging strand synthesis uncoupling, poor processing of 5’-flaps, secondary DNA structures and R-loops are additional challenges for replication machinery when replicating telomeric sequences, which can lead to the formation of double-stranded breaks, thus, making these replication impediments triggers for BIR [146,148,149,150,361,362,363]. Template switching during ALT may involve the sister telomere or telomere from another chromosome (see Figure 5A). Alternatively, as it is discussed [50,228] ALT-dependent telomere elongation may occur through extension of the D-loop within T-loop possibly involving many iterations of DNA invasions and DNA slippage (see Figure 5B). Telomere length can also be extended via invasion of the 3’- end of the telomere into an ITS accompanied by formation of the ITL (see Figure 5C). Multiple invasions and/or DNA slippage may also contribute to the extent of telomere elongation in the later case. Interestingly, this pathway might provide the source of DNA for telomere extension even if sister or other telomeres are not available for invasion or are critically short. 

DNA helicases and replication fork remodelers, which facilitate strand-exchange reaction, homologous duplex invasion, template switching, and subsequent branch migration steps, can influence the initiation and the outcome of BIR-dependent telomere elongation and are essential for ITSs instability. We found that expansions of the Ytel repeats depended on the Rad6/Rad5 pathway of postreplicative repair of DNA as well as on Rad51 and Rad52 HR proteins and Srs2 and Mph1 helicases [300]. Rad5 is a critical TS factor in yeast. This helicase associates with native telomeres and is essential for telomere length regulation and maintaining the viability of pre-senescent cells [364,365]. Deletion of the *RAD5* gene decreases instability of poly(GT) tracts in yeast [366]. Srs2 and Mph1 are the two DNA helicases and anti-recombinases which are involved in remodeling of replication forks and D-loops and implicated in telomere maintenance including the ALT process. The yeast Mph1 helicase contributes to template switching during BIR and can promote telomere uncapping and accumulation of ssDNA at telomeres when overexpressed, which results in premature senescence in the absence of telomerase [367,368]. The very same proteins play essential role in telomere maintenance and are implicated in the ALT process. Two Rad5-related DNA translocases in human cells, HLTF and SHPRH, are tumor suppressors which prevent genomic instability [369,370]. RTEL1 (Regulator of Telomere Elongation helicase 1), the functional analog of Srs2 in human cells, associates with the replisome through binding to PCNA and plays a crucial role in telomere and genome-wide replication [371]. Specifically, RTEL1 is required to repress the fragile telomere phenotype and is recruited to telomeres through TRF1 [120]. Human and mouse cell lines bearing mutated or inactivated RTEL1 produce a large excess of extra-chromosomal circular telomeric DNA (T-circles) generated by the improper resolution of T-loops [372,373,374]. The human ortholog of the Mph1, the FANCM protein associated with Fanconi Anemia, plays a role in the replication stress response, stabilization of replication forks and replication fork restart [128]. A recent study shows that FANCM is required for efficient replication at ALT telomeres [375]. Rad51 and Rad52 are the two central proteins of homologous recombination that mediate the strand exchange reaction and are involved in the ALT process (see Section 1.4). Their involvement in ITS’s instability further corroborates the idea that terminal and interstitial telomeric repeats share similar mechanisms leading to their instability and length alteration (Figure 5D). An exciting prediction from this idea is that ITSs length destabilization can be an indicator of activated ALT.

Our latest data suggest that protein factors directly interacting with telomeric repeats are central in determining their instability pathways. For instance, human-like telomeric (Htel) tracts (TTAGGG)n placed in our *URA3-Intron* model system also expanded at a high rate. However, the properties of Ytel and Htel repeats and the mechanisms underlying their instability were different. Expansions of Htel repeats didn’t depend on the Rad6, Rad5, or Rad51 and Rad52 proteins but occurred as a result of replication slippage by DNA polymerase ε and depended on factors influencing chromatin modification and chromatin remodeling. We found that the Tbf1 protein (see Section 1.3.2) binds to interstitial Htel repeats and recruits HDACs, which ultimately impedes DNA replication leading to repeat instability (Aksenova and Radchenko, preliminary data).

## 3. Conclusions

ITSs have been viewed for a long time as a junk DNA associated with chromosomal rearrangements and aberrations. Data accumulated during the last decade altered our understanding of these genomic elements. They interact with the nuclear membrane and are involved in the formation of ITL-loops, telomere maintenance, and genome-wide regulation of gene expression and 3D genome structure [266,267,297,329,330]. These functions may explain conservation of some ITSs in distinct lineages and their relative abundance in eukaryotes. While ITSs are located inside chromosomes, they can behave as bona fide telomeres. ITSs bind telomere-specific proteins such as components of Shelterin complex, which then regulate their functioning and stability. ITSs present adjacent to DSBs regulate localization of broken ends inside the nucleus and the outcome of their repair [335]. Chromosome breakage within ITS traсts may lead to the appearance of a new telomere or to the inversion of the chromosomal arm [299,336]. ITSs are dynamic elements of the genome as they undergo significant length variation. The high rate of ITS expansions could explain their length polymorphism within an organism as well as their amplification in many species. Tight parallels were observed between the mechanisms of ITSs expansions and the mechanisms of ALT [300] (see also Figure 5).

The dynamic nature of ITSs seem to stem from their interference with the DNA replication process. ITSs are covered by telomere-binding proteins, e.g. Shelterin complex components, that affect the progression of the replication fork through them. Thus, binding of the TRF1 protein is required to stabilize the common fragile 2q14 ITS site in human cells [285]. At the same time, our data suggest that the binding of the Rap1 and Tbf1 proteins - yeast Shelterin counterparts - to ITSs results in the replication roadblock. The resultant genetic instability depends on the strength of this roadblock. Simple slowing of the replication fork provokes DNA strand slippage resulting in repeat expansions. Strong roadblocks lead to the fork collapse, DSB formation and their subsequent repair via BIR and TS. The latter pathways have arisen in prokaryotes to restart stalled or collapsed replication forks. Given the intrinsic ability of simple DNA repeats to anneal “out of register,” these pathways are implicated in the large-scale expansions of many microsatellite repeats including telomeric repeats and trinucleotide repeats (TNRs) [376]. Existence of these mechanisms and their co-evolution with simple DNA repeats likely facilitate establishment of functional repetitive sequences in the course of genome evolution, such as telomeres and centromeres. The existing complex regulatory pathways of telomere maintenance involving ALT could be a result of evolution of these primary mechanisms.
